# Toxicity of Neem Seed Oil against the Larvae of *Boophilus decoloratus*, A One-Host Tick In Cattle

**DOI:** 10.4103/0250-474X.58191

**Published:** 2009

**Authors:** M. K. Choudhury

**Affiliations:** Department of Pharmaceutical Chemistry, School of Pharmacy, Addis Ababa University, Addis Ababa, Ethiopia

**Keywords:** Neem seed oil, *Azadiracta indica*, Meliaceae, *Boophilus decoloratus*, *ixodidae*, tick, *in vitro* toxicity

## Abstract

The *in vitro* toxicity of neem seed oil (*Azadirachta indica* A. Juss, family: Meliaceae, *Dogon yaro* in Hausa language in Nigeria) was tested against the larvae of a one-host tick, *Boophilus decoloratus* (family: *Ixodidae* or hard tick, commonly known as blue tick) parasitic mainly to cattle generally found in savannas of tropical equatorial Africa. The 20, 40, 60, 80 and 100% concentrations of neem seed oil were found to kill all (100% mortality) the larvae after 27, 27, 27, 27 and 24 h respectively.

The neem (*Azadirachta indica* A. Juss, family: Meliaceae) is a very popular tree in most tropical and sub-tropical regions of the world. It is known as neem all over the world and *Dogon yaro* in Hausa language, widely spoken in Nigeria. Neem is a hardy tree that grows vigorously in desert areas and in a harsh climate. It possesses versatile medicinal properties along with antibacterial and antifungal activities. The people of India have been using neem leaves for centuries for various aspects.

Neem has proved effective against certain fungi that infect the human body and are difficult to control by synthetic fungicides. Neem preparations showed toxicity to 14 common fungi, including members of the following genera. *Trichophyton* (an athlet's foot fungus that infects hair, skin and nails), *Epidermophyton* (a ringworm that invades both skin and nails of the feet), *Microsporum* (a ringworm that invades hair, skin and rarely nails), *Trichosporon* (a fungus of the intestinal tract), *Geotrichum* (a yeast-like fungus that causes infections of the bronchi, lungs and mucous membranes) and *Candida* (a yeast-like fungus that causes infections in mouth, vagina, skin, hands and lungs)[1]. The toxicities of neem seed oil against the larvae of *Amblyomma variegatum* (a three-host tick)[2], *Rhipicephalus sanguineus* (a three-host tick)[3] and *Boophilus decoloratus* (a one-host tick)[4] were published earlier. *Boophilus decoloratus*, the blue tick is a one-host tick occurs throughout the Ethiopian region and Southern Asia especially in humid areas. It is parasitic chiefly on cattle and equines, but also found on sheep and goats, wild ungulates and dogs. The engorged females have a slaty blue colour and pale yellow legs. The females lay around 2500 eggs[5]. It transmits the disease Babesia bigemina known as *Babesiosis* (rise in temperature to high fever, inappetence, red urine, anaemia, jaundice, emaciation, death)[6]. There is a great economic loss in livestock production today due to infestation of ticks in animals in the world. The present article describes the toxicity of neem seed oil against the larvae of *Boophilus decoloratus* collected from cows.

The kernels were collected from the ground under the neem tree in the Ahmadu Bello University campus, Zaria, Nigeria, depulped, washed with water and dried under sun. The kernels were crushed using a crucible and blender, then boiled with water for 1 h when the oil separated and floated to the top. The two phases were separated using a separating funnel and the oil, a viscous liquid was collected from the upper phase and dried over anhydrous Na_2_SO_4_.

The larvae of a one-host tick,*Boophilus decoloratus* were used in this study. The engorged female ticks were collected from Zangon Shunu area in Zaria, Nigeria by hand picking from the bodies of the cows. Each engorged female tick was placed in a clean plastic tube plugged with cotton wool and incubated at 29° with relative humidity of about 80%. The eggs laid in each tube were transferred into a clean tube with cotton plug and dead female ticks discarded. The eggs were kept under same incubating conditions until they hatched into larvae and then starved in the incubator at 29° for two weeks before use.

Petri dishes (9 cm diameter) with one sheet of filter paper inside of the same size were used for the experiment. Five different concentrations of neem oil, 100, 80, 60, 40 and 20% were used for the study. One milliliter (100%), 0.8 ml (80%), 0.6 ml (60%), 0.4 ml (40%) and 0.2 ml (20%) of neem oil were evenly soaked (spread with a spatula) on different filter papers of each Petri dish. Distilled water soaked in filter paper was used as a control (0% concentration). Then different numbers of larvae were put inside each plate with the help of a soft brush and then monitored at certain interval of time. The number of larvae found dead in each plate was recorded and the results were summarized in Table 1.

**TABLE 1:** MORTALITY OF LARVAE AT DIFFERENT CONCENTRATIONS OF NEEM SEED OIL AND DIFFERENT TIME

Plate No	Concentration of neem oil	Number of larvae taken	Number of dead larvae after		
			
			3 h	24 h	27 h
1	0% (water)	160	0 (0%)	0 (0%)	0 (0%)
2	20%	76	6 (8%)	57 (75%)	76 (100%)
3	40%	96	10 (10%)	60 (63%)	96 (100%)
4	60%	103	10 (10%)	44 (43%)	103 (100%)
5	80%	110	12 (11%)	91 (83%)	110 (100)%
6	100%	154	149 (97%)	154 (100%)	

From the results obtained, it is clear that the mortality of larvae was concentration and time dependent. With 0% concentration of neem oil (control), there was no mortality at any time. 100% mortality was observed with 20, 40, 60, 80 and 100% concentrations after 27, 27, 27, 27 and 24 h, respectively. Thus the neem oil can be safely used for the control of ticks in animals due to its non- adverse effect in animals. Moreover, the neem oil with both fungicidal and bactericidal properties could treat these infections caused from the bite of ticks. The oil is easily available for its use as an inexpensive herbal medicine. This result will be very much useful for the farmers all over the world.
